# From a View of the Hospital as a System to a View of the Suffering Patient

**DOI:** 10.3389/fpubh.2021.800603

**Published:** 2022-01-07

**Authors:** Gillie Gabay, Smadar Ben-Asher

**Affiliations:** Achva Academic College, Arugot, Israel

**Keywords:** hospital directors, patient-centered care, salutogenics, suffering, tertiary public hospitals, values of care, narrative, vignette

## Abstract

**Purpose:** Hospitals aspire to provide patient-centered care but are far from achieving it. This qualitative mixed methods study explored the capacity of hospital directors to shift from a hospital systemic-view to a suffering patient-view applying the Salutogenic theory.

**Methods:** Following IRB, we conducted in-depth narrative interviews with six directors of the six Israeli academic tertiary public hospitals, focusing on their managerial role. In a second meeting we conducted vignette interviews in which we presented each director with a narrative of a suffering young patient who died at 33 due to medical misconduct, allowing self-introspection. Provisional coding was performed for data analysis to identify categories and themes by the three dimensions of the sense-of-coherence, an anchor of Salutogenics: comprehensibility, manageability, and meaningfulness.

**Results:** While at the system level, directors reported high comprehensibility and manageability in coping with complexity, at the patient level, when confronted with the vignette, directors acknowledged their poor comprehensibility of patients' needs and patient's experience during hospitalizations. They acknowledged their poor capacity to provide patient-centered care. Meaningfulness in the narrative interview focused on the system while meaningfulness in the vignette interview focused on providing patient care.

**Conclusions:** The evident gaps between the system level and the patient level create lack of coherence, hindering the ability to cope with complexity, and are barriers to providing patient-centered care. To improve the delivery of patient-centered care, we suggest ways to consolidate the views, enabling the shift from a systemic-view to a patient-view.

## Introduction

Hospitals are complex multi-agency systems that need to act as single entities in synergy with many subsystems and elements that are dependent on one another and use multiple operating variables (1). The successful management of such complex systems requires skilled managerial thinking that places at its center both the hospital as a system and the patients (2). Since hospitals provide care to suffering patients, the research questions this study addresses are: How can the suffering of an individual patient be part of the system's complex operational considerations? Can a hospital director, who oversees the whole system and its operations, see the suffering of the individual patient from the third room on the right in the internal, orthopedic, or oncology department, within the big picture of the hospital? Is the hospital director acquainted with the picture of the individual patient? Does the director recognize the patient's special story as part of his or her immediate responsibility for the operation of the hospital as a whole system, rich with factors, components, and immanent tensions? These questions are the focus of this study.

The Institute of Medicine (IOM) (3) defined patient-centered care (PCC) as an essential domain of quality care (2). PCC entails emotional support, physical comfort, communication, education, continuity of care, coordination of care, family involvement, and access to care (4–6). Moreover, PCC dimensions include patient-clinician concordance, meeting patient expectations, integrating patients within the environment, viewing a patient as a person, conducting dialogue and interaction, sharing patient experience, and documentation of the patient's narrative (7). PCC fosters healing relationships; responds to patients' emotions; engages patients in informed and collaborative decision making; and seeks to provide each patient with care she values (8, 9). PCC leads to improved health outcomes through improved patient safety, higher patient trust in physicians, higher adherence, better recuperation, fewer readmissions, and higher quality of life (7, 8, 10–12).

PCC must be implemented, as a moral imperative at hospitals, to improve the quality of care and patient well-being (13–16). However, since hospital directors cope with complexity and uncertainty, they may often have little awareness of the values underlying the hospital mission in daily routines, resulting in a failure to achieve the organizational vision of PCC (1, 17). With the rise in life expectancy and the expansion of chronic illness, PCC is a desired approach that emphasizes not only improved clinical outcomes but also good patient experiences (8). PCC is what patients expect and what hospitals, physicians, and hospital directors should be remembered for (8, 17). And yet, achieving PCC in hospitals around the world is challenging (5, 18, 19).

How may hospital directors fulfill their operational responsibility for the hospital as a system and still see the patient experience as the cornerstone of optimal care; as of paramount importance to patients; as a strategic asset; and as a driver of reputation for both patients and insurers, forming part of PCC implementation? To address PCC, hospital directors need to understand patients' individual needs, perspectives, and values, and to translate this understanding into decision-making (20). The most frequent complaints of patients, however, are that their concerns are neither heard nor understood (8, 21). Directors who have a medical background have a better understanding of clinical challenges, know how to effectively communicate with medical professionals, and lead to higher: efficiency; bed occupancy; safety; operating margins; market share; resources management, and financial performance and to fewer surgical infections (22–26).

These strengths may result in their capacity to better bridge the divide between cure, care, and administration. At the same time, directors with a medical background (DMB) experience strong tensions in prioritizing needs of patients over systems' efficiency (27, 28). The tensions that DMBs experience may be due to the challenging shift from a systemic view of the hospital to a view of the patient as an individual with emotional needs (29). DMBs experience both high job-engagement and a strong sense of responsibility. Job engagement enriches their meaning, zest, and vitality in life. A high sense of responsibility among DMBs, however, can lead to job-related overload, fatigue, risk of burnout, and an inability to see the suffering of an individual patient. The inability to see the patient's suffering may shape decisions that ineffectively implement PCC (8, 15). To achieve PCC, it is essential that DMBs become aware of and assimilate the experiences and needs of suffering patients during lengthy hospitalizations and that they make decisions to manage these experiences and needs, as part of their complex demanding role (10).

### The Current Study

This qualitative study responds to previous calls to investigate how DMBs, who are responsible for numerous managerial duties, can simultaneously understand the clinical task and see patients as individuals (24, 29–32). We investigate the capacity of DMBs to shift their focus from the systemic hospital-level view to the view of the suffering of an individual patient and back to the hospital-level view, which now encompasses the needs and experiences of patients. This study explores: (a) the capacity of DMBs to simultaneously see the suffering of the patient and the needs of the hospital as a system; (b) the challenges involved in this capacity; and (c) the operational implications of this capacity in the context of PCC implementation. The goal of the study is to broaden our understanding of how DMBs process experiences of suffering patients; to identify barriers to the resolution of tensions among DMBs that may inhibit PCC achievement; and to suggest ways to resolve these tensions.

### The Theoretical Framework

The Salutogenic theory (33–35) focuses on important resources that generate well-being in the presence of stressful conditions, termed salutary factors. The salutary factors comprise the sense of coherence (SOC), which is a central asset in understanding the world one lives in; one's ability to manage the complexity of this world; and the ability to grasp its meaning, as elucidated below. The Salutogenic model is a strong theoretical formulation that has been applied in health and in hospitals and in the community to promote patients' well-being and to promote nurses' well-being in a mental hospital (36–38). It has recently been applied to promote social ties between people in the community to promote well-being (39). Most theories of resilience were developed in the context of terror and in natural disasters. We adopted Antonovsky's theory of resilience, as it targets the health context pertaining to both patients and medical professionals, based on Antonovsly's experience as a hospital physician who focused on the range between pathology and health (40). We posit that the Salutogenic model may be employed among DMBs who make decisions that have an immense impact on patients' well-being. The delivery of PCC requires the integration between biomedical- physiological elements and emotional elements intertwined holistically and existentially (3, 41).

*Comprehensibility*, the cognitive dimension, refers to the extent to which one perceives internal and external stimuli as rationally understandable, and perceives information as orderly, coherent, clear, and structured rather than chaotic, disordered, random, unexpected, and unexplained (33). *Manageability*, the behavioral dimension, is defined as the degree to which one feels that she or he has resources to meet the requirements of the stimuli one is bombarded by (33). The third dimension, *meaningfulness*, the motivational dimension, is the extent to which one feels that life has an emotional meaning, that problems are worth one's commitment and dedication, and are challenges rather than burdens. Meeting and coping with the requirements entail one's motivation to solve the problems creating stress and one's willingness to invest energy to solve the problems and find meaning in being able to manage the situation (33).

The delivery of PCC is associated with patient experiences of preserving self-esteem together with clinical characteristics of care involving clinician-patient relationships, support, and empowerment (42). High SoC promotes the implementation of PCC through an integration of biomedical care with psychosocial care (15). SoC promotes PCC through positive self-identity; increased tolerance for various feelings; awareness of the other, enabling a multi-perspective view; nurturing; reassurance of self-worth; a climate of unconditional positive regard; empathy and genuineness; and emotional, psychological, and social well-being (43). The uniqueness of this study is in applying the Salutogenic model in the design of action strategies for hospital directors in the context of PCC (35). This study responds to previous calls to study the Salutogenic model among health professionals (44, 45).

## Materials and Methods

### Ethics

Ethical approval (IRB #099) was granted by the Ethics Committee of the Research Board at the Academic Institution with which the first author was affiliated (to be disclosed upon acceptance). Participants gave their written informed consent to participation and to publication of parts of the interviews.

### Participants

Participants were six DMBs (4 men and 2 women) head directors of all six academic tertiary public general hospitals in Israel, with diversity in the hospitals' location and in medical specialty. Despite the small sample size, participants fulfill the requirement for having a wide arrange of attributes (gender, age, hospital size, and location) (46). Participants' ages ranged from 50 to 70 years.

### Recruitment

We recruited participants by contacting the district managers of the six tertiary academic Israeli hospitals, asking for DMBs' participation in a study on the experience of DMBs. All DMBs agreed to participate despite being extremely busy, which was possibly an indication of the importance of their mission. A brief phone conversation was held with each DMB before the interviews to describe the study goals. The nature of the research question, its complexity and scope, and the scarce knowledge on this topic make the size of this rare sample of head directors of tertiary hospitals, acceptable for an explorative study (47). All identifying demographics at the individual level were omitted to ensure anonymity and confidentiality (48).

### Procedures

The first author introduced herself as a health management researcher from an academic institution studying hospitalizations. She assured the participants of anonymity and that they could stop the interviews at any point they chose. Participants signed a written informed consent regarding participation and publication (48). The first author thanked them for their willingness to contribute to our understanding and explained the study methodology. Twelve interviews were conducted, two with each director off hospital premises. The first interview was a 90–120 min narrative interview regarding the daily experience of the DMBs. The second interview presented DMBs with a vignette and asked for their response.

Vignettes are an accepted qualitative research tool encompassing short depictions of typical scenarios that are intended to elicit responses revealing values, perceptions, and impressions among interviewees (49). Interviewees are typically asked to reflect and respond to presented scenarios, typically personal stories (50). Within qualitative research, vignettes are valuable for exploring people's perceptions, beliefs, and meanings about sensitive situations, tapping elements that may not be readily accessible through other means of inquiry (50–53). The use of vignettes provides a less intrusive and non-threatening way of obtaining perceptions, opinions, beliefs, emotions, and attitudes, based on responses or comments of observers to whom the vignette is presented (51).

A 30–45 min vignette interview, was aimed at presenting the narrative of a suffering young oncological patient and asking each director to respond to the vignette. This combination of qualitative techniques made it possible to expose DMBs both to the view of the hospital as a system focusing on their role, in the narrative interview, and to the view of the individual patient, in the vignette, allowing self-introspection. Interviews were audio-taped, transcribed verbatim, and translated into English. The first author assigned each participant a number (1–6). After transcribing the interviews, each participant received a copy of the interviews and approved their content.

### The Narrative Interview

As typical for narrative interviews, to allow participants to discuss the topic in their own words and free of constraints from fixed-response questions, one general, open-ended question was asked (54): “What has been your daily experience at the hospital since you were appointed as director?” This interview aimed at understanding the daily experience of hospital directors, i.e., their conscious, processed perceptions regarding their managerial role in a complex system. The first author aimed at sending a message of acceptance of everything participants said, verbally and non-verbally, by active listening and awareness of body language so that messages of comfort and acceptance were created. Five days to a week following the in-depth interview, the first author conducted a second interview applying the vignette technique.

### The Vignette Interview

The first author chose to complement the first interviews with a vignette interview to evoke intra-personal questions and introspection among DMBs regarding patient suffering. Since the vignette technique is a less threatening medium, it enabled DMBs to comment on a patient's narrative rather than directly discuss the failure of clinicians to provide quality care (55). To prevent defensiveness, the first author stated that the wrongdoing of a resident leading to a patient's paralysis and death occurred in a hospital just like the one that the directors manage but not in the hospital with which they are affiliated. Furthermore, reading the vignette allowed DMBs to define the situation at their own pace, providing them a sense of control and at the same time, openly eliciting perceptions, beliefs, moral codes, and attitudes in response to the vignette of a suffering patient. The responses of DMBs to the vignette may have predictive power, reflecting how they behave when they experience a similar real-life event (55). Thus, by complementing the narrative interview with a vignette, we sought to gain insights into emotions and raw, unprocessed perceptions of DMBs of a patient's suffering in their daily routine as DMBs.

Each DMB read the same seven-page written anonymous continuous narrative of a 30-year-old oncology patient depicting the patient's experience through a series of stages (55). The narrative in the vignette was plausible, in a context well-understood by DMBs, internally consistent, and not too complex (52). The vignette depicted the cancer trajectory of a young patient who was a successful manager in a high-tech firm, from diagnosis through lengthy hospitalization until his death at the hospital. The patient suffered greatly due to loss of his self-esteem, to his disenfranchised grief emanating mostly from clinicians' attitudes and lack of humanistic care and conduct, greatly intensifying the suffering caused by the illness (10). The vignette was based on five narrative interviews depicting the patient's experience, beginning with false diagnoses, a correct diagnosis of a malignant cancer, successful complex surgery, failure of a resident to listen to the patient complaining about immense post-surgical pain, consequent paralysis thereafter, and a 2-year hospitalization until the patient's death. The vignette was selected as a complementary technique to “wind down” from the rational response presented through DMBs' processed perceptions in the narrative interview to their response to the patient's experience (56). When DMBs completed the reading of the vignette and looked ready to respond, the first author asked: “What emotions and gut feelings did this narrative evoke in you as you were reading it?” Participants were guided to focus on their emotions rather than on clinical tasks and share their view as a DMB, who is responsible for realizing the hospital's vision as well as for encountering patients' suffering.

### Data Analysis

We followed five data-driven analytical steps: we read and re-read each of the narrative interviews; identified important themes and elements in the data; encoded these elements prior to interpretation by the SoC dimensions; we identified theme co-occurrence; and searched for relationships among aspects (35, 57). We coded the raw data deductively according to the three specific dimensions of SoC (57, 58). Thematic analyses were used to identify and describe ideas within the data that capture complexities of meaning within textual data (58). Moving on to the analysis of the vignette interviews, we identified and analyzed responses grounded in the experiences of the patient; experiences of the DMB, both as a director and as an individual. We related the subjective experience of the DMBs—their perceptions, feelings, frustrations, and experiences—to capture intricacies of meaning within the data through implicit and explicit elements of resilience in providing care (59). Based on our epistemology, we used provisional theory-driven analysis for pinpointing, exploring, and recording themes within each category (dimension) of data in the interviews (58, 59).

Analyzing *the comprehensibility* dimension, we sought data referring to: What DMBs understand and what they don't understand; DMBs' impressions regarding the experience of suffering patients; and the DMBs' feelings as they processed the vignette. Analyzing the *manageability* dimension, we sought data referring to DMB's coping with traumatic events of patients and with providing PCC. A prerequisite for coping with a stressful situation is that one can understand it to some extent; what is comprehensible is easier to manage. Analyzing the *meaning* dimension, we sought data referring to: DMBs' feelings through their daily tasks—are they performed out of a sense of deep satisfaction, or out of distress? When a DMB thinks about their life, do they ask why they exist at all? What is the DMB's life mission?

To enrich the analysis, we addressed gaps between the narrative interview and the vignette interview across overt and covert messages relating to resilience at the systemic level (Narrative) and at the patient level (Vignette). An analysis of the gaps between interviews across dimensions allows an informed analysis between declared role aspects of SoC and intrapersonal aspects of SoC, which exist within one's mind. Since the response was spontaneous and DMBs were unaware of the three aspects of SoC and were unfamiliar with the suffering narrative, such an analysis may improve our understanding of the challenges in delivery of PCC.

### Quality Criteria

We maintained general quality standards of thematic qualitative research. We acknowledged our theoretical positions and values regarding the research issue. To support transferability of findings, we described the methodology of the study in detail. To assure rigor, we maintained the following quality criteria: clarification and justification; procedural rigor; representativeness; saturation of data; a thick description of the phenomenon; interpretative rigor; coding based on unstructured interviews; reflexivity; evaluative rigor; inter-rater reliability; and transferability (48, 59, 60). Assigning statements into dimensions of SoC reflects, to some extent, interpretations of researchers who are operating within a cultural-historical context. Due to their subjectivity, in some situations different qualitative researchers may differ slightly in assigning statements to dimensions (61). Therefore, only statements about which there was complete agreement between researchers regarding the dominance of assignment into dimensions (comprehensibility, manageability, and meaningfulness) were included in the categorization into dimensions.

## Results

Findings of both narrative and vignette interviews are presented through the three SoC dimensions: comprehensibility, manageability, and meaningfulness, as they emerged from data analysis.

### Narrative Interview

#### Comprehensibility

During the narrative interviews, DMBs spoke in a confident, authoritative tone characteristic of professionals who are in control of the field at issue. They incisively presented their overall perception, responsibility for the realization of the hospital vision, responsibility for operations, and achievements since they were assigned as directors. They shared how their position connects policy with patients' needs, their perception of humanistic values underlying the delivery of care, and the importance of continued professional development of clinicians toward providing optimal care.

The DMBs attributed great importance to clinical knowledge. They described the work at the hospital as an outflow of knowledge and management skills that, at times, serve as mechanisms of control as well: “*Control is the name of the game” (*DMB 4*)*.

The DMBs often used the words “explain,” and “teach,” believing in their ability to drive professionalism at the hospital and viewing medicine as a science that is subject to a multitude of factors with no scientific answers to all issues. At the same time DMBs emphasized the organizational culture of protocols and commitment to fulfilling guidelines as defined by a DMB: “To avoid mistakes and damages” (DMB 6).

The DMBs describe various control measures that provide a sense of control over the system despite uncertainties “I work with supporting methodologies of advanced computing and appropriate technologies” (DMB 4). Knowledge management is an important component in DMB's sense of manageability. One DMB introduced a three-level typology of management: operational, training, and guidance with the highest level as leadership: “I was asked to outline an annual work plan in 45 s. I gave a 5-year work plan in 3 min” (DMB 2). The DMBs presented an understanding of values of care, such as compassion, human dignity, empathy, and concern for a patient, as a strong organizational pillar to which each employee is committed: “Values of altruism and mission should' flow through the bones” (DMB 5). To gain an in-depth understanding of a patient's needs, DMBs who supervise the clinical care meet with the patient and based on knowledge they gather, provide the optimal care plan. The DMBs claimed that knowledge is always partial as previous diagnostic knowledge may be missing, and post-discharge from the hospital, there may be new variables that become part of the clinical picture. Despite describing the significant power of doctors for the patients and their illness, the DMBs still believe that the patients are most knowledgeable about their own body. DMBs emphasize the asymmetry of power as undermining patient engagement or respect for the right of autonomy, which are pillars of PCC. Asymmetric power in doctor-patient relationships inhibits PCC: “The clinician has the knowledge; the patient only has the body” (DMB 6). This key insight brought DMBs to present their main position, that physicians must work in collaboration and must communicate with the patients, from the initial encounter with them through their discharge from the hospital. “In practice, the hospital does not provide information to families and to patients, resulting in conflicts with family members, particularly when there is a need to make decisions” (DMB 2); “We need to make the family an assistant and not an auditor” (DMB 4).

#### Manageability

To describe his daily work, one of the DMBs used the metaphor of the orchestra conductor: “*As a hospital director, you conduct a multi-participant orchestra where you depend on each of its arms for your ongoing functioning”* (DMB 2). DMBs expressed a shared view that the metrics of success are financial metrics rather than metrics that provide an advanced integrated medical care: “*In the end, the success measure of the good manager is whether or not she or he succeeds in financially balancing the hospital”* (DMB 2).

In Israel, all DMBs (including women) served in the military, either for the three mandatory years or for an extended military career in the medical corps. They replicate the militaristic terminology of battlefields, in the management of civilian hospitals: “*I know the military as a commanding management system;* (DMB 1); *I feel like I'm in the battlefield”* (DMB 6); “*This is a never-ending craft, where you constantly have to fight this war*” (DMB 4). DMBs agreed that control measures are pivotal to hospital management. Control measures which were perceived as unclear or as inhibiting the high quality of care were seen as inhibiting manageability: “*We come to work to provide care but feel that the impossible bureaucracy of the system puts us in a position where we must bypass it to provide care”* (DMB 4).

DMBs view management as not only referring to how they run the hospital but also to the way they manage medical professionals. They viewed development of the medical staff, especially of young physicians, is a managerial skill to which DMBs attribute great importance: “*Sometimes a resident makes a diagnosis that clinical leaders have not yet made. While a single doctor is good, a team is much better”* (DMB 1). DMBs viewed the priorities that senior physicians set as their own responsibility, although the senior physicians are the ones in charge of the day-to-day professional functioning. The DMBs viewed the existing measures as not closely related to the clinical tasks that physicians supervise. DMBs attributed impediments in realizing the hospital vision to internal and external factors that inhibit manageability: “*There is insufficient staffing and insufficient ongoing medical education from the student level to the senior physician level”* (DMB 5). The directors' need for managerial control goes beyond control of the organizational system to the professional field they supervise, which includes the patient-physician interaction. Because they are aware of their inability to be present when patients are provided with care, they express frustration associated with incidents that are beyond their control.

#### Meaningfulness

Meaningfulness emerges from comprehensibility and manageability. For DMBs, meaningfulness is the belief that their actions are important and that they are committed and responsible for realizing the hospital vision. Beyond a sense of pride, DMBs mentioned two elements that enhance their meaningfulness: managerial success reflected in outcome-measures and satisfaction derived from caring for patients. One DMB described the hospital director as the leader: “*Leadership at the hospital is the essence of management”* (DMB 3). “*The director has charisma, builds trust, and is followed by medical professionals.”* (DMB 1).

To sum up, DMBs have a high sense of competitiveness and assess themselves as able to manage a complex system that presents them with new challenges every day. They spoke confidently, demonstrating high self-control. The image of an orchestra conductor demonstrates their view of the system as being conducted around three axes: the managerial axis of the hospital system, the professional medical axis, and the axis of overseeing processes of care and relationships with patients. With respect to the Salutogenic approach, DMBs appear to perform highly across the dimensions of resilience, providing them with an unquestionable experience of professionalism and high performance of their managerial role. Although they are aware of their limitations in the field of knowledge and management, their overall experience is one of competitiveness and meaningfulness.

Will the DMBs sense of confidence and high achievement remain stable when they face the vignette of a suffering patient that reflects a failure of the medical operating system and of physicians in processes of care which lead to his death at the age of 30? To explore this question, we returned to the DMBs for the vignette interview in which we presented the narrative of a suffering patient.

### Interview II—The Vignette of the Suffering Patient and DMBs' Responses to It

The DMBs read the narrative of the suffering patient with great engrossment. Upon completing their reading, the interviewer observed that their body contracted and stiffened, they seemed to freeze in place, the flow of words stopped, their eyes wandered to the window separating the office from the outside surroundings. They seemed engaged in introspective contemplation. The patient now had a name, parents, young children. They stared at the printed narrative on their desk right in front of them. The DMBs fell silent for several minutes that seemed longer. When two of them finally met the first author's eyes again, they expressed feelings of sadness, “It is so sad.” “they said quietly,” “It's so painful and so frustrating.” The first reaction of most DMBs after reading the vignette was emotional.

Analysis of the verbal responses of DMBs to the vignette reveal intensified frustration, deepening some issues they raised in the narrative interviews. The DMBs did not refer much to the many medical errors that physicians made during the patient's diagnosis and during the lengthy hospitalization. Instead, they mainly referred to the physicians' attitudes toward the patient, judging the physicians' priorities. The exposure of DMBs to the vignette and their authentic responses to the patient narrative contradicted their sense of knowing, deliberate protocols, clear vision, 5-year plans, and feeling like leaders in full control as they navigate the hospital from chaos to certainty. The narrative of the patient in the vignette moved DMBs from potency and control to helplessness. As in the analysis of the narrative interview, here too, we analyze the DMBs' responses using the three dimensions of SoC.

#### Comprehensibility

DMBs expressed helplessness about their sense of comprehensibility of the experiences of patients in hospitalizations demonstrating their lack of resilience:

“*What do I understand as a director? [Quiet]…If anything, I understand that I do not really understand the true needs of patients. I do not understand what the patient needs beyond the clinical treatment. What are our weaknesses? What are the gaps? I don't understand*” (DMB 1); *Communication is poor; we don't know how to talk with patients, and we spend less time with them”* (DMB 4).

There was criticism of the lack of direct personal contact with the patient: “*This relationship is of great importance and has no substitute”* (DMB 5). The DMBs questioned the decision-making process for patients and expressed fear of the potential harm to patients stemming from failures of the medical professionals.

DMBs attributed some failures to the breach of patient-trust in physicians, when the latter prefer to “align” their conduct with interests of the system, that are, many times, inconsistent with needs of patients:

“*A dangerous mistake kills*” (DMB 1); “85% of guidelines are completely unnecessary because they do not guarantee or improve quality of care; they just take away responsibility from the regulator” (DMB 3); “Machine-based knowledge does not seem to be satisfactory where meeting the patient and applying humanistic understanding is required.” (DMB 2)

DMBs critique internal guidelines while ignoring the fact that all hospital guidelines are their own responsibility:

“*Criteria and medical supervision result in unnecessary treatments”* (DMB 5);“*If there is a severe case of medical error, at worst, it is the lack of attention and negligence that must be addressed but usually, this is due to the complexity of care”* (DMB1).

Failure is perceived as a medical error or negligence on the part of clinicians, but it is not directly or indirectly attributed to managerial processes or to their own responsibility as supervisors monitoring these processes: “*The human factor is our problem. We have training sets and simulators but our impact on their character is small” (DMB 4)*.

#### Manageability

DMBs anxiously pointed out that many of the medical professionals, particularly physicians, do not adhere to humanistic values of care in their daily work: “*Compassion, human dignity and empathy are empty words”* (DMB 5); “*Mistakes kill, but the doctor does not see that and thinks he is half God in a white robe”* (DMB 2). “

DMBs' shared their frustration with their lack of confidence in the judgment of physicians and the way the system works creating failures in providing PCC:

“*There is a lack of sensitivity, there are errors in judgment, there is a horrible depersonalization”* (DMB 6); “*There is great damage. His medical condition worsened which reflects the broken medical system, literally broken”* (DMB 5).

Another DMB criticized the lack of direct personal contact with the patient: “*This relationship is of great importance and is irreplaceable”* (DMB 5);

The DMBs emphasized their cynicism as expressing their loss//lack of faith in the system. The cause seems to be the difficulty of instilling values of mission that are, in DMBs' view, the basics of medicine: “*At 16:00 young doctors in their first year of residency strip off their uniforms and exit the hospital, leaving their stethoscope behind. It is legal but it frustrates me …”* (DMB 4). While in the interview the managerial aspect took center stage, in the vignette interview the managerial aspect was pushed aside. DMBs hardly responded to the suffering narrative from a managerial point of view and responded by describing their frustration and by the place of values in patient care.

#### Meaningfulness

The DMBs connected meaningfulness with values of care:

“*We are about the most precious thing we all have – life itself” (DMB 3); “Human dignity in hospitals must be a priority”* (DMB 1); “*I think this is the most important system in the country”* (DMB 2).

The DMBs view physicians as role models for compassion and respect for human dignity. In the DMBs' view, physicians are required to listen to the patient while demonstrating compassion, sensitivity and accountability. The DMBs expressed frustration when they were faced with the vignette which exposes the lack of such values of care in encounters and interactions with patients. The DMBs identified with the patient's suffering, especially since they were asked to relate to their gut feelings:

“*More than anything, I am frustrated by doctors who are disconnected and non-empathetic towards the patient”* (DMB 2); “*My obvious feeling is helplessness and personal-professional disappointment that I cannot really help. Patients need something that I can't give them”* (DMB 6); “*What we are missing is the human spirit and dignity.”* (DMB 3); “*This situation causes me to be cynical”* (DMB 4); “*I am very sad. We fail to respect and preserve the patient's human dignity and privacy. When the patient is at the most serious and fragile moment of illness,”* (DMB 5).

The DMBs openly criticized the conduct of clinicians regarding the patient's experience:

“*I think the patient experience is very poor”* (DMB 4); “*I felt empathy and a great deal of sorrow”* (DMB 2); “*In long and continuous hospitalizations, one encounters the suffering. You are exposed to layers of suffering over suffering, and it is not heroic”* (DMB 3).

The DMBs conveyed the message that while it is extremely important that the patient-doctor relationship be based on respect, dignity, privacy, and the uniqueness of each patient, they became aware of the discrepancy between the vision and the reality. DMBs felt competent in themselves but dissatisfied with medical professionals. Responding to the narrative, DMBs related to the guiding values:

“*In actuality, there is lack of empathy, lack of compassion, lack of respect for human dignity, empathy and compassion between staff, and patients do not exist”* (DMB 5); “*We are impatient and a patient with complex comorbidity does not receive respect and privacy”* (DMB 5); “*I would like the doctor to tell the patient ‘Sorry you are waiting; we are crowded today”* (DMB 3);

The DMBs view relationships with patients as very gratifying:

“*There is utmost delight and great professional satisfaction in caring for patients. There is a sense of pride – doing the most advanced medicine in the world; it brings great pride”* (DMB 3); “*I think that in such a unique, dedicated profession, it is most important to establish trust”* (DMB 2).

DMBs hinted that they suppressed unpleasant feelings through cynicism:

“*The extent of our cynicism is the extent of our frustration. I encounter cynicism; it's another world that also describes my experience and comes from the field. Physicians are cynical*” (DMB 5). “*In the end, no matter what tremendous efforts I invest in staff development and in quality of care, at the end, there is one rotten apple that spoils the barrel”* (DMB 6).”

To sum up, it seems that DMB's share the view that strengthening core values of medical care in physician-patient relationships is part of their managerial role. Despite the complexity of the situation, DMBs were honest and understood the disparity between the problems and what they spoke of in the interviews. It is important to intervene to enhance DMBs' awareness of patients' experiences enabling a multi-lens view of patients, physicians, and the system.

**Figure 1:**
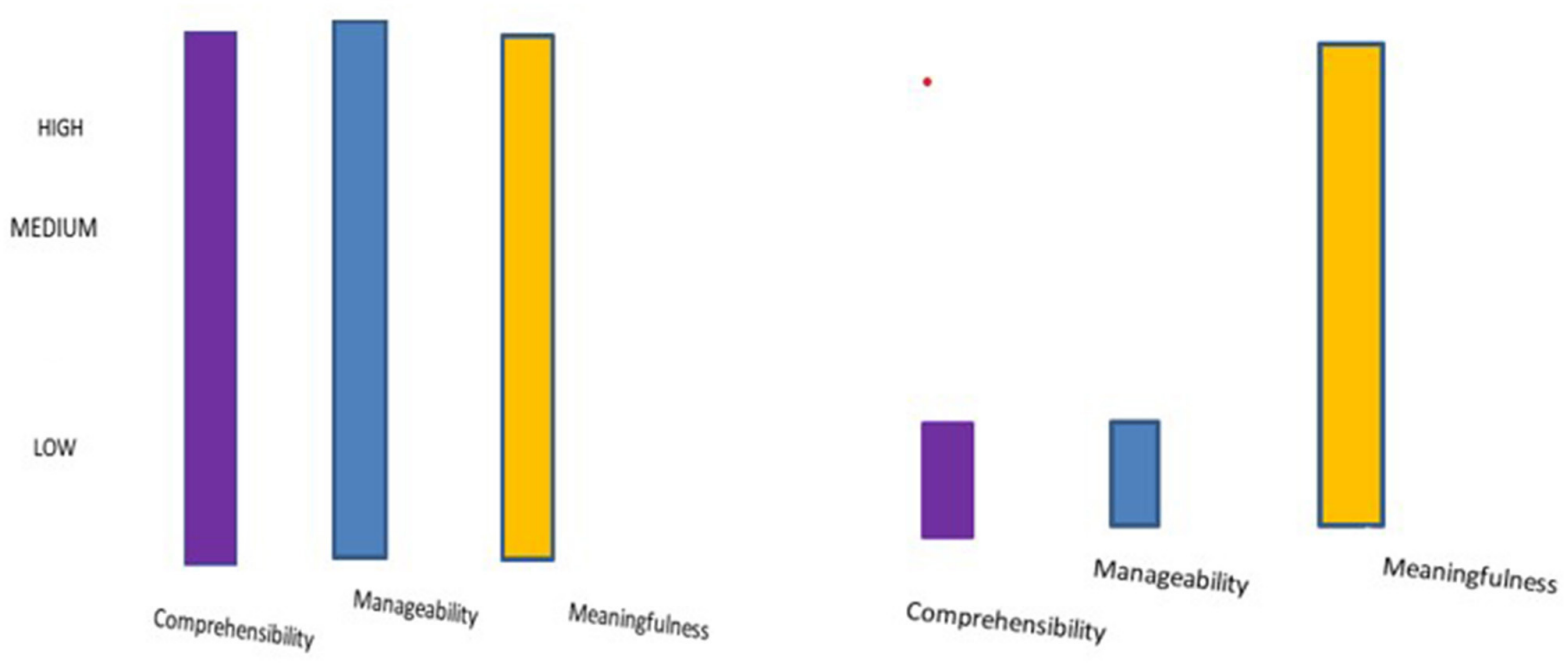
Dimensions of sense of coherence in the narrative and vignette interviews.

Figure 1 presents the dynamics of SoC dimensions in the narrative interviews and the vignette interviews.

## Discussion

This study explored the capacity of DMBs to shift from a view of the hospital as a system to a view of the individual patient's suffering at tertiary hospitals in the context of PCC. This research project makes theoretical, methodological, and practical contributions. Theoretically, to our best knowledge, this study is the first to apply the Salutogenic framework to hospital directors. Second, this study reveals a gap between DMBs' strong SoC at the system level and poor SoC at the patient level, thus extending the knowledge on barriers to achieving PCC in hospitals. Methodologically, this study is the first to integrate two qualitative methods, a narrative interview with a vignette interview to complement the managerial perspective of DMBs with the intra-personal perspective of DMBs regarding patient care. Practically, since findings revealed two separate realities, one reality at the system level and one reality at the patient level, we suggest interventions to consolidate these realities into one through strengthening the SoC of DMBs and enhancing their capacity to shift between the system view and the patient view while coping with the high-stress, complex environment of the hospital. A higher SoC will facilitate decision making that considers emotional needs and experiences of patients, resulting in improved outcomes (7, 32, 62).

### What Is the Capacity of DMBs to Simultaneously See the Suffering of the Patient and the Needs of the Hospital as a System—Gaps Between SoC in Each Interview

In the narrative interviews, DMBs shared their self-perception of their high manageability of the system despite its complexity. DMBs reported that although they must operate at a rapid pace in a highly dynamic environment, they perceived systemic-hospital processes as comprehensible, highly manageable, and a great source of pride and meaningfulness. Thus, the high level of SoC is demonstrated by DMBs' perception of complexity as comprehensible, manageable, and meaningful. A high level of SoC also entails the individual's self-assessment that she or he not only comprehends the complexity, but also has the required resources to cope with complexity which further motivated them to cope. This finding echoes a previous study that found that healthcare providers focus mostly on knowledge when delivering care and their attitudes are shaped through their responsibilities and roles (63). In the narrative interview, DMBs emphasized their high understanding and control of the system, understanding of the humanistic values of care and of being responsible for their assimilation among all employees, and understanding of processes of managing professionals. According to the Salutogenic framework, there is a direct relationship between one's level of SoC and one's ability to mobilize cognitive and emotional resources to cope effectively with stressful situations.

Confronting DMBs with the vignette of a suffering patient brought them to acknowledge their poor level of SoC regarding their comprehensibility of patients' experiences and needs and their poor manageability in providing PCC.

Therefore, their capacity to mobilize cognitive and emotional resources to cope with patients' needs is limited. The emotional vignette bypassed DMBs' direct cognition—exploring not only what DMBs said when presenting their processed perceptions of reality but also the meaning of what they said. They acknowledged the lack of humanistic care and the poor patient care; they critiqued the priorities of physicians' and their self-perception; they acknowledged the medical errors; and expressed concerns over the damage to both patients and the hospital. As the DMBs responded to the vignette, despite their position of authority and power, they perceived their hands as tied and felt helpless.

The DMBs moved from high potency to helplessness. This pendulum may have caused the DMBs to experience frustration, role conflict, and distress. Role- distress may create tensions that have been manifested in high psycho-physiological activity (32, 34–37), perhaps inhibiting implementation of PCC. The gap between the narrative interviews and the vignette interviews supports a previous study that viewed PCC as a unique emergent ethical stance which accords salient relational ethics encompassing respect, engagement, and embodied knowledge (7).

### What Are the Challenges in Shifting From a System View to a Patient View?

In the vignette interview, the DMBs' both non-verbal and verbal responses to the narrative of suffering reflected sadness, sorrow, and frustration. The DMBs realized that their understanding of patient's needs, emotions, and experiences in lengthy hospitalizations is poor. They realized that empathy, compassion, and viewing the patient as an individual are lacking, and “humanistic values are empty words.” The response to the narrative of the suffering patient in terms of manageability was an authentic response of lack of control, helplessness, inability to shape the characteristics of doctors, and despite the aspirations to provide PCC, inability to provide patients with PCC. Contributors to resilience from the DMBs' perspective related mainly to routines, protocols, and high financial performance. These findings suggest a consistency between resilience and manageability at the system level. Thus, while essential protocols organize priorities and structure the reality at the hospital, it is questionable whether this tight structuring contributes to resilience and to achieving PCC.

While the narrative interviews reflected high comprehensibility and high manageability of the complexity, the vignette interviews reflected poor comprehensibility of patients' needs and experiences, resulting in poor ability to cope and provide PCC. Low comprehensibility and poor manageability reflect a chasm between the vision of the hospital and its implementation. Meaningfulness in the narrative interview focused on the system as DMBs viewed themselves as competent leaders. Meaningfulness in the vignette interview focused on the patient, as DMBs viewed themselves as responsible for patients' lives. Gaps in SoC between interviews may reflect a pattern of high “doing” and low “being” among DMBs and may reflect two separate realities, one of the directors and the systems they manage and one of hospitalized patients. This pattern of two separate realities inhibits the promotion of PCC and may deepen the chasm between the hospitals' vision of PCC and the capacity of DMBs to realize this vision, providing patients with less-than-optimal care. While DMBs accept the existent reality, patients, hoping to receive empathetic, compassionate, high-quality care would most probably view these gaps as inhibiting their safety, rather than satisfying their need for respectful encounters with clinicians at the hospital.

The narrative of the suffering patient was emotional, entailing reflection, subconscious, and an inner dialogue at a time of an acute crisis, but when the DMBs spoke about their view of the suffering narrative, they used mostly cognitive channels rather than emotional channels. The DMBs borrowed terms from the managerial, cognitive domain rather than from the emotional, healing domain: “*I wish we had an X-ray of patients' feelings, thoughts, and emotions”* (DMB 2). Responding cognitively to the emotional suffering of a patient may reflect a displacement of DMBs' emotions resulting from differences in cultural conventions which underlie decision-making (64). The cultural convention of DMBs may be analytical and rational, leading to the use of artifacts from medicine vs. the cultural convention of patients in crisis, which entails emotions. Gaps between climatic cultural conventions may lead to reductionism in decision-making rather than a multi-perspective view that considers the emotional needs of patients, inhibiting PCC.

Since qualitative research points at the quality criteria as a precondition for generalization, the quality criteria of this research enables the generalization of our findings to DMBs and hospital directors, in public hospitals, across the world, who are experiencing both the chasm between the aspired PCC and the hospitals' incapacity to achieve PCC and the tension of integrating the view of the hospital as a system with the view of suffering patients as individuals.

### What Are the Operational Implications of the Incapacity to Shift From a System View to a Patient View for PCC Implementation?

Meaningfulness is an inspirational force, sustaining meaning, zest, and vitality (39, 65). By Salutogenics, people may explore their attitudes in a situation from multiple perspectives: those of the hospital processes and those of patients, physicians, and suffering patients. Findings suggest that DMBs, in their cultural conventions, are unaware of the patient's perspective. To successfully implement PCC, the DMBs' operational focus and patient-centered focus must be bridged. DMBs are called upon to broaden their perspective in decision-making by developing a stronger SoC that may enable them to better implement PCC, while preserving managerial energy and maintaining their own well-being (32, 34–37). As part of strengthening comprehensibility, it is important to help DMBs identify their challenge of shifting from an operational managerial view to a patient level and the stressors it entails (66). Balanced comprehensibility, manageability, and meaningfulness at the patient level, not only at a system level, are essential to improving DMBs' capacity to shift from a system view to “seeing” the individual patient in implementing PCC. Since SoC develops over an entire life cycle, DMBs may strengthen their SoC as part of an advanced program of continued education.

### Where Do We Go From Here? Practice Implications for Bridging the Gap

Hospital care must move beyond ideology and declared values to an evidence-based PCC (67). The implementation of PCC should be across all levels, from compassionate clinicians who employ strategies of compassion to decision making of hospital directors. Schibbye and Thornye (68) introduced “the art of wondering” which promotes acceptance, empathy, concern, and authenticity (42). The art of wondering corresponds to emotional skills acquired through SoC development (34), which may take place at the individual level or at the group level. A previous study on the journey of professionals into themselves suggests that the group is key to creating a climate of social and emotional connection (69). Participant-oriented methodologies such as dialogues or discussion groups for reflection of DMBs on their own practice were found to be effective (70–72). One pivotal emotional skill in SoC development is reflection (34). Reflection is a complex process requiring time for introspection about the meaningfulness of the DMB's role in the life of a patient. It is important to reflect on what the DMB is doing, why, and how it affects suffering patients and PCC. Reflection may enhance DMBs' self-sensitivity and self-awareness, promoting clarity about one's circumstances. DMBs may strengthen their SoC to avoid over-zealous attention to their administrative duty. Exploring and reflecting on their role-tensions is a continuous reflective task that considers how the two perspectives—that of the system's and that of the patients'—complement each other. Interventions such as personal executive coaching, monthly analysis of patient narratives upon their discharge from the hospital, and discussing insights with supervising clinicians, may sharpen sensitivities of individual clinicians and DMBs to the integration of the systemic view with the patients' view.

DMBs are called upon to enhance patient trust (73). Patient trust may be enhanced by exploring the patient's crisis, the emotions it evokes, and the resources, support, and communication behaviors of clinicians that may alleviate the patient suffering that emerges from the conduct of clinicians. Similar to psychoeducation interventions for patients to enhance their knowledge about illnesses (71), psychoeducation for DMBs can enhance DMBs awareness of patients' experiences during hospitalization. When comprehensibility for patients' experiences is established, DMBs' attitudes and actions may change, integrating the psychosocial aspects in processes of care. Managerial decisions that improve patients' experiences will enhance DMBs' capacity to provide PCC (70, 74). DMBs may focus on “doing” and “being” simultaneously and understand the reciprocal feedback-loops among them (75). Feedback loops between the managerial reality of the DMB and the reality of the suffering patient will validate the patient as an important stakeholder and may help DMBs develop reflexivity as second nature. An additional emotional skill in developing SoC is habitual self-tuning (76, 77).

Self-tuning develops existential meaning, which is important for work-related well-being (9, 39). When DMBs become adept at “stimulus” in self-tuning, introspection will become habitual in the form of their ability to read and interpret their physical and emotional signals and the signals from their surroundings (e.g., patients and physicians) (76). Other emotional skills in SoC development are empathy and social responsibility, which lead to competence in supporting suffering patients and in developing physicians (38, 77). The use of vignettes of patients' narratives may also serve to sharpen DMBs' insights. Through introspection, similarly to the process in the vignette interview, DMBs' sensibilities may provide relevant and useful reflection processes, which in turn, will broaden the perspective and the range of actions enabling DMBs to make relevant adaptations to hospital processes that correspond with patients' needs.

### Strengths, Limitations, and Future Studies

Research exploring the capacity of DMBs to resolve tensions emanating from the shift between a system view and a view of the suffering patient is scarce. Our use of the vignette method enabled us to bypass emotional barriers and defensiveness among DMBs, revealing the chasm between aspirations for humanistic values in care and the hospital vision, on the one hand, and poor patient experiences on the other hand. Some novel, interesting insights are made by this study, but it has its limitations. Conducting narrative interviews at only one time, rather than conducting a longitudinal study with additional interviews 6 months and a year following the initial interviews, inhibits learning regarding the application of the insights DMBs may have gained in implementing PCC. Future qualitative studies may continue this line of research with large balanced samples, in other countries. where the cultural background differs from the one in this study. Future studies may also investigate differences between the management styles of female DMBs and those of male DMBs.

## Conclusions

An idealization of the systemic view without understanding the experience of a suffering individual patient may jeopardize the ability to crystallize priorities that facilitate PCC. Focusing on the system level leads DMBs to partial data, on the basis of which they shape their views of the world. Lack of data among DMBs regarding the experience of an individual patient may lead to a misinterpretation of the hospital as a whole and may distort their perception of causes of poor patient-centered care. DMBs undertake leadership by inspiring others while promoting values and vision. Our Salutogenic analysis sheds light on a weakness of DMBs who are very proficient at the system level but do not have adequate skills and capacities when it comes to individual patients and their distress. Any training strategy that aims at developing Salutogenic capacity should be grounded in the ontological stance that Salutogenesis represents: a continuous learning process of “doing” and “being.” Training that aims at a stronger SoC of DMBs is essential to implementation of PCC and considering the patient's distress in DMBs' decision-making. Policymakers and DMBs are called upon to reallocate resources for the expansion of Salutogenic skills among DMBs. Avenues to promote this change may include peer training workshops, personal coaching and participation in a human spirituality program where reflection and introspection are enabled through poetry, philosophy, or theater.

## Data Availability Statement

The original contributions presented in the study are included in the article/supplementary material, further inquiries can be directed to the corresponding authors.

## Ethics Statement

The studies involving human participants were reviewed and approved by IRB #99 the Ethical Board of the College of Management Academic Studies, Israel. The patients/participants provided their written informed consent to participate in this study.

## Author Contributions

GG: conceptualization, data collection, data analysis, and writing the first draft. SB-A: data analysis, reviewing the first draft, and creating the figure. All authors contributed to the article and approved the submitted version.

## Conflict of Interest

The authors declare that the research was conducted in the absence of any commercial or financial relationships that could be construed as a potential conflict of interest.

## Publisher's Note

All claims expressed in this article are solely those of the authors and do not necessarily represent those of their affiliated organizations, or those of the publisher, the editors and the reviewers. Any product that may be evaluated in this article, or claim that may be made by its manufacturer, is not guaranteed or endorsed by the publisher.
