# Corrigendum: From a View of the Hospital as a System to a View of the Suffering Patient

**DOI:** 10.3389/fpubh.2022.898656

**Published:** 2022-04-11

**Authors:** Gillie Gabay, Smadar Ben-Asher

**Affiliations:** Achva Academic College, Arugot, Israel

**Keywords:** hospital directors, patient-centered care, salutogenics, suffering, tertiary public hospitals, values of care, narrative, vignette

**Incorrect Author Name**

An author name was incorrectly spelled as **Smadar Ben Asher**. The correct spelling is **Smadar Ben-Asher**.

The authors apologize for this error and state that this does not change the scientific conclusions of the article in any way. The original article has been updated.

## Publisher's Note

All claims expressed in this article are solely those of the authors and do not necessarily represent those of their affiliated organizations, or those of the publisher, the editors and the reviewers. Any product that may be evaluated in this article, or claim that may be made by its manufacturer, is not guaranteed or endorsed by the publisher.

